# Systemic thrombolysis with newer thrombolytics vs anticoagulation in acute intermediate risk pulmonary embolism: a systematic review and meta-analysis

**DOI:** 10.1186/s12872-023-03528-w

**Published:** 2023-09-29

**Authors:** Don Mathew, Susmitha Seelam, Karandeep Bumrah, Akil Sherif, Utsav Shrestha

**Affiliations:** 1 Department of Internal Medicine, University of Pittsburgh Medical Center (UPMC), Pittsburgh, PA USA; 2 Department of Cardiology, St Vincent Hospital, Worcester, MA USA; 3 Department of Pulmonary and Critical Care Medicine, West Virginia University, Morgantown, WV USA

**Keywords:** Intermediate risk PE, Meta-analysis, Systemic thrombolysis, Anticoagulation

## Abstract

**Background:**

Randomized controlled trials (RCTs) comparing systemic thrombolysis to anticoagulation in intermediate risk pulmonary embolism (PE) have yielded mixed results. A prior meta-analysis on this topic had included studies that used lower than standard dose of thrombolytics and included thrombolytic agents that are no longer available. Hence, interpreting the findings of that paper is not valid in contemporary practice.

**Objectives:**

We undertook a systematic review and meta-analysis of randomized controlled trials of systemic thrombolysis with newer thrombolytic agents vs anticoagulation in intermediate risk PE.

**Methods:**

This systematic review and meta-analysis is reported according to the Preferred Reporting Items for Systematic Reviews and Meta-Analysis (PRISMA) statement.

**Results:**

Nine randomized controlled trials were included in the study. We did not find any difference in in-hospital mortality (RR: 0.79; 95% CI: 0.42–1.50; I^2^: 0) or risk of major bleeding (RR:2.08;95% CI: 0.98–4.42; I^2^: 23.9%) between systemic thrombolysis and anticoagulation. Systemic thrombolysis was associated with lower risks for vasopressor use (RR: 0.27; 95% CI: 0.11–0.64, I^2^: 0) and secondary/rescue thrombolysis (RR: 0.25; 95% CI: 0.14–0.45; I^2^: 0). But systemic thrombolysis was found to have an increased risk of intracranial hemorrhage (RR: 4.55; 95% CI: 1.30–15.91; I^2^:0). There was no difference in mechanical ventilation between the two groups (RR: 0.61; 95% CI: 0.31–1.19, I^2^:0).

**Conclusion:**

In our meta-analysis of randomized controlled trials of systemic thrombolysis vs anticoagulation in intermediate risk PE, we did not find any difference in in-hospital mortality or overall risk of major bleeding. With systemic thrombolysis, we found lower risks for vasopressor use and need for secondary/ rescue thrombolysis and an increased risk of intracranial hemorrhage.

**Supplementary Information:**

The online version contains supplementary material available at 10.1186/s12872-023-03528-w.

## Introduction

Acute venous thromboembolism is the third leading cause of cardiovascular mortality following myocardial infarction and stroke [1]. Acute pulmonary embolism (PE) occurs when an embolus breaks off a thrombus, which often develops within the leg or pelvic veins and occludes a blood vessel of the pulmonary artery tree [2, 3]. In the US, the incidence of PE is between 1–2 in 1000 and is responsible for around 300,000 deaths annually [4]. Acute pulmonary embolism can cause right ventricular (RV) failure leading to hemodynamic collapse and death [5]. Acute RV failure, which results from impaired RV filling and/or reduced RV flow output is a critical determinant of severity. Risk stratification and early institution of treatment is important in suspected cases of acute PE. The European Society of Cardiology stratifies PE into high risk, intermediate risk, and low risk. High risk (Massive) PE involves patients who are hemodynamically unstable, and in these patients systemic thrombolysis (ST) is recommended. Anticoagulation (AC) is recommended in cases of PE that are not high risk [6]. The treatment of patients with acute PE who are hemodynamically stable but demonstrate signs of RV dysfunction (intermediate-risk) is anticoagulation, but randomized controlled trials that compared systemic thrombolysis to anticoagulation in this group have yielded mixed results. A prior meta-analysis that studied the effects of systemic thrombolysis vs anticoagulation in intermediate-risk PE patients found mortality benefit with systemic thrombolysis but at increased risk of major bleeding and intracranial hemorrhage [7]. But the results of this analysis cannot be applied to contemporary practice for the following reasons.

- They included trials that used older thrombolytic agents such as streptokinase and urokinase that are not used in contemporary medical care in the U.S. In fact, these agents are no longer available in the U.S.
- Trials that used lower than standard dose of thrombolytics were included. Using studies that used variable dosing for systemic thrombolysis questions the validity of the findings.

Due to these reasons, we sought to perform a systematic review and meta-analysis of systemic thrombolysis vs anticoagulation in intermediate-risk PE patients including studies that used standard dosing of newer thrombolytic agents.

## Methods

This systematic review and meta-analysis is reported according to the Preferred Reporting Items for Systematic Reviews and Meta-Analysis (PRISMA) statement [8].

### Study outcomes

The primary outcomes of interest were in-hospital mortality and major bleeding as defined by the International Society of Thrombosis and Hemostasis (ISTH) [9]. The secondary outcomes were mechanical ventilation, secondary/rescue thrombolysis, vasopressor use and intracranial hemorrhage. For inclusion, the study should have reported at least one primary outcome. Only studies that included intermediate risk were considered. For our study, Intermediate risk PE was defined as cases with acute PE with objective evidence of RV dysfunction but hemodynamically stable. Evidence of RV dysfunction included positive CT or echo findings and/or elevated cardiac biomarkers.

### Study selection

We included randomized controlled trials on adult population that compared systemic thrombolysis to anticoagulation in acute intermediate-risk pulmonary embolism.

Cases without RV dysfunction were not included in this study. Studies where PE grade was not assessed were not considered. Only studies that used standard dose of thrombolysis were included in the study. Studies that used lower dose of thrombolytics and studies that used catheter directed methods for delivery of thrombolytics were not included.

### Search strategy

**Fig. 1:**
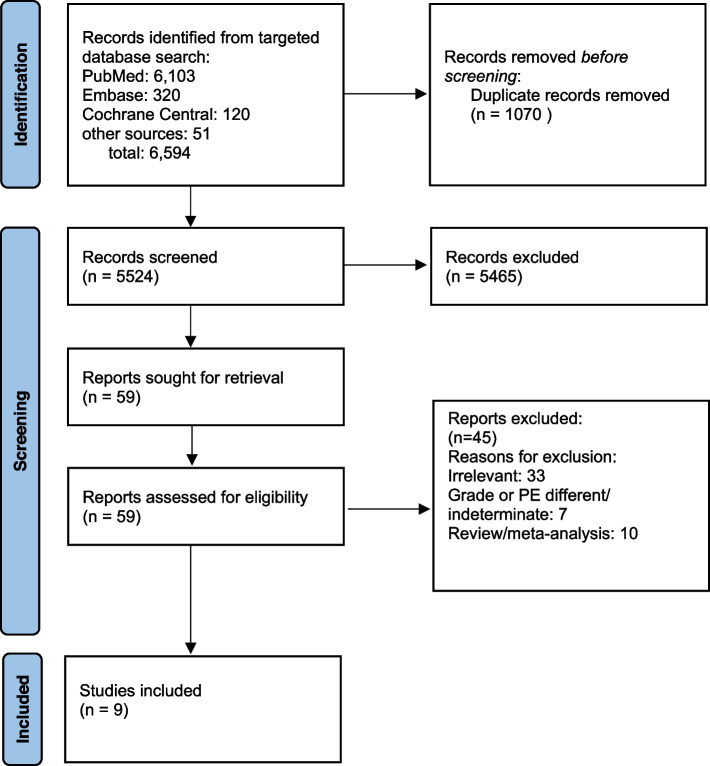
PRISMA Flow diagram

A targeted literature search was conducted on PubMed/MEDLINE, EMBASE and Cochrane CENTRAL for articles from inception until March 21, 2023. The search was conducted using a combination of keywords and MeSH terms: ("Pulmonary Embolism"[MeSH Terms] OR "high-risk pulmonary embolism"[All Fields] OR "intermediate-risk pulmonary embolism"[All Fields] OR "acute submassive pulmonary embolism"[All Fields]) AND ("anticoagulant agent"[All Fields] OR "anticoagulants"[MeSH Terms] OR "heparin"[All Fields] OR "heparin"[MeSH Terms] OR "low molecular weight heparin"[All Fields] OR "heparin, low molecular weight"[MeSH Terms] OR "fibrinolytic agent"[All Fields] OR "fibrinolytic agents"[MeSH Terms] OR "thrombolytic therapy"[MeSH Terms] OR "alteplase"[All Fields] OR "tenecteplase"[All Fields] OR "tenecteplase"[MeSH Terms] OR "tissue plasminogen activator"[All Fields] OR "tissue plasminogen activator"[MeSH Terms] (Supplemental File). Search was restricted to adult patients. No language restrictions applied. Grey literature was reviewed by screening scientific research proceedings, conference abstracts and clinical trials registered on clinical trials.gov. The study selection process is shown in Fig. 1.

### Data extraction and quality assessment

Two authors (S.S and K.B), both attending physicians in Internal Medicine, independently screened and reviewed articles. Duplicates were identified on EndNote and removed manually. Disagreements between reviewers were resolved by discussion and achieving consensus.

Quality of studies and reporting bias were assessed using the Cochrane risk of bias assessment tool [10].

### Data synthesis and analyses

Analysis was conducted in R, version 4.1.2, using the statistical package “metafor” [11]. Risk ratios were determined using a random effects model generated by the DerSimonian and Laird (DL) method if the number of studies included in the analysis was larger than three [12]. The DerSimonian-Laird method with the modified Hartung-Knapp-Sidik-Jonkman variance correction was used as a sensitivity analysis of the DerSimonian-Laid method [13, 14]. We used a fixed-effects method based on the Mantel–Haenszel method if the number of studies was three or fewer [15]. Results were reported in 95% Confidence Intervals and depicted using Forest Plots. Study heterogeneity was tested using a formal χ ^2^ test with a Q – statistic and quantified using Higgins I ^2^ statistic. A I^2^ = 0 was considered to indicate no heterogeneity, values of I^2^ as < 25%, 25–75%, and > 75% to indicate mild, moderate and high degrees of heterogeneity, respectively [16]. As we included studies spanning over three decades, we conducted a meta-regression to assess if publication year was an influencing factor. Meta-regression was also conducted using mean patient age, gender, and presence of cancer as variables to determine if they had an effect on primary outcomes. Publication bias was not evaluated as the number of studies was less than ten [17].

## Results

The study selection process is given in Fig. 1. Nine randomized controlled trials met inclusion criteria and were included in our analysis [18–26]. Characteristics of included trials are given in Table 1.

**Table 1:** Study Characteristics

Author year	Thrombolytic	Anticoagulant	Risk of Bias	In-hospital mortality	Major Bleeding
Levine 1990 [23]	Alteplase	Heparin	Low	ST (1/33) AC (0/25)	ST (0/33) AC (0/25)
Dalla-Volta 1992 [24]	Alteplase	Heparin	Low	ST (2/20) AC (1/16)	ST (3/20) AC (2/16)
Goldhaber 1993 [25]	rtPA	Heparin	Low	ST (0/46) AC (2/55)	ST (2/46) AC (0/55)
Konstantinides 2002 [20]	Alteplase	Heparin	Low	ST (4/118) AC (3/138)	ST (1/118) AC (5/138)
Becattini 2010 [18]	Tenecteplase	Heparin	Low	ST (0/28) AC (1/30)	ST (2/28) AC (1/30)
Fasullo 2011 [19]	Alteplase	Heparin	Low	ST (0/37) AC (5/35)	ST (2/37) AC (1/35)
Meyer 2014 [21]	Tenecteplase	Heparin/LMWH/ fondaparinux	Low	ST (6/506) AC (9/499)	ST (58/506) AC (12/499)
Kline 2014 [22]	Tenecteplase	LMWH	Low	ST (1/40) AC (1/43)	ST (1/40) AC (0/43)
Sinha 2017 [26]	Tenecteplase	Heparin	Low	ST (12/45) AC (2/41)	ST (1/45) AC (1/41)

*LMWH* Low-molecular-weight heparin, *rtPA* recombinant tissue plasminogen activator, *ST* Systemic thrombolysis, *AC* Anticoagulation

Risk of bias was assessed using the Cochrane risk of bias assessment tool [10]. Studies were graded based on randomization process, deviation from intended intervention, missing outcomes, measurement of outcomes, and selection of reported results. All included studies were found to be of good quality with low overall risk of bias (Supplemental Fig. 1).

### Primary outcomes

#### In-hospital mortality

**Fig. 2:**
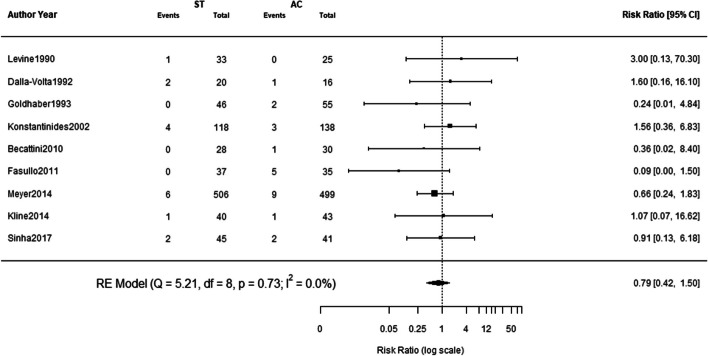
In-hospital Mortality Risk Figure legend: ST = systemic thrombolysis; AC = anticoagulation; RE = random effects; CI = confidence interval

There was no significant difference between systemic thrombolysis and anticoagulation on in-hospital mortality. The pooled risk ratio for in-hospital mortality with systemic thrombolysis was 0.79 (95% CI: 0.42–1.50; I^2^: 0; *p*-0.47). Forest plot of in-hospital mortality risk is given in Fig. 2.

### Major bleeding

**Fig. 3:**
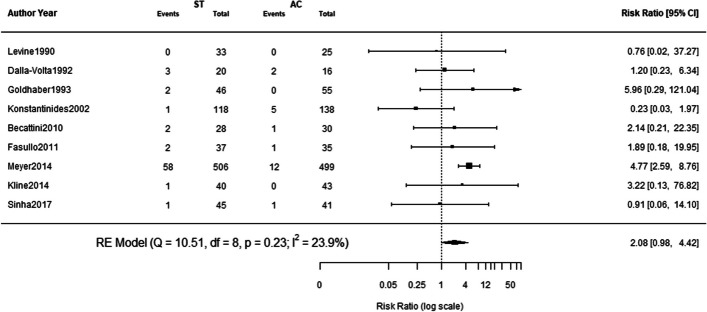
Risk of Major Bleeding Figure legend: ST = systemic thrombolysis; AC = anticoagulation; RE = random effects; CI = confidence interval

We did not find any significant difference in risk of major bleeding between systemic thrombolysis and anticoagulation. The risk ratio for major bleeding with systemic thrombolysis was 2.08 (95% CI: 0.98–4.42; I^2^: 23.9%; *p*-0.06). Forest plot of risk of major bleeding is given in Fig. 3.

### Sensitivity analysis

We found similar results from sensitivity analysis. The risk of in-hospital mortality was 0.79 (95% CI: 0.43–1.45, I^2^: 0; p-0.39) and major bleeding was 2.08 (95% CI: 0.94- 4.56, I^2^: 23.87%; *p*- 0.06).

### Meta-regression

Year of publication, mean patient age, gender, and presence of active cancer were included in meta-regression analyses to evaluate the effect of these variables on primary study outcomes. None of these variables were found to have a significant effect on in-hospital mortality or major bleeding [ In-hospital mortality: p(publication year) = 0.3, p (age) = 0.5; p(male) = 0.3, p (cancer) = 0.4; Major bleeding: p (publication year) = 0.4, p (age) = 0.6, p (male) = 0.8, *p* (cancer) = 0.8].

### Secondary outcomes

#### Mechanical ventilation

We did not find any difference in mechanical ventilation between systemic thrombolysis and anticoagulation (RR: 0.61; 95% CI: 0.31–1.19, I^2^:0; *p*-0.15) (Supplemental Fig. 2).

#### Vasopressor Use

Systemic thrombolysis was associated with significantly lower risk for vasopressor use (RR: 0.27; 95% CI: 0.11–0.64, I^2^: 0; *p*-0.003) (Supplemental Fig. 3).

### Secondary/rescue thrombolysis

Systemic thrombolysis was associated with significantly lower risk for secondary/rescue thrombolysis (RR: 0.25; 95% CI: 0.14–0.45; I^2^: 0; *p*- < 0.0001) (Supplemental Fig. 4).

### Intracranial hemorrhage

We found a higher risk of intracranial hemorrhage with systemic thrombolysis (RR: 4.55; 95% CI: 1.30–15.91; I^2^:0; p-0.02) (Supplemental Fig. 5).

## Discussion

In our meta-analysis of systemic thrombolysis vs anticoagulation in intermediate-risk PE, we did not find any difference in mortality or overall risk of major bleeding. We did find an increased risk of intracranial hemorrhage, but the result was not precise due to the wide confidence interval. This is likely because the available studies except for the PEITHO trial by Meyer et.al were small sized and hence were likely underpowered to detect any differences. We found decreased risk for vasopressor use and need for secondary/rescue thrombolysis with systemic thrombolysis. This demonstrates the effectiveness of systemic thrombolytic therapy to prevent hemodynamic decompensation in intermediate-risk PE.

To identify patients at risk of decompensation, the European Society of Cardiology (ESC) has further stratified intermediate risk PE into intermediate high risk and intermediate low risk groups. Intermediate high-risk PE includes patients who are hemodynamically stable with PESI class III-V or sPESI >  = 1, RV dysfunction evident by TTE or CT and elevated cardiac troponin levels. Intermediate Low risk includes hemodynamically stable patients with PESI class III-V or sPESI >  = 1 but with either one or none of the objective evidence of RV dysfunction (imaging with TTE/CT or troponin). They recommend closer monitoring of intermediate-high risk PE patients due to the higher risk of hemodynamic decompensation in these patients [6].

The ESC guidelines currently recommend anticoagulation for acute treatment of intermediate-risk PE. They recommend rescue thrombolytic therapy for patients who develop hemodynamic deterioration on anticoagulation treatment [6]. The findings from our study offer further evidence to support this recommendation. Post hoc analysis of clinical trial and registry data have linked heart rate > 100, BP 90–100 mm Hg, respiratory rate > 20/min, SaO2 < 90% and presence of chronic heart failure and active cancer to be associated with disease severity and increased risk of deterioration in normotensive patients, but there are no studies to date that have confirmed that patients with any combination of these risk factors would benefit from upfront reperfusion therapy. In the UK, the National Early Warning Score 2 (NEWS2) is recommended to monitor for clinical deterioration, but so far there is no formal recommendation to use any scoring system for PE patients [27]. As an alternative to rescue thrombolytic therapy, surgical embolectomy or catheter-directed treatment is recommended for patients with hemodynamic deterioration on anticoagulation treatment [6].

Catheter based techniques are touted to accomplish the benefits of systemic thrombolysis i.e. preventing hemodynamic decompensation but without its bleeding risks. But so far there is only one small scale RCT to date that compared catheter directed thrombolysis with anticoagulation [28]. A large scale RCT between ultrasound assisted catheter directed thrombolysis and anticoagulation is ongoing [29]. Administering lower dose of thrombolytics is proposed as another alternative and has been shown to be effective in a small RCT [30]. A large scale RCT comparing lower dose thrombolysis to anticoagulation is also ongoing currently [31]. Due to lack of high-quality evidence, primary reperfusion with catheter directed therapy is not recommended as first line treatment in neither intermediate nor high risk PE. The current recommendation is that they should be considered in hemodynamic deterioration despite anticoagulation (treatment failure) and in failure of systemic thrombolysis. Mechanical thrombectomy should be considered if there are contraindications to systemic thrombolysis [27].

Our study offers further evidence to support the current ESC guidelines in the management of acute intermediate risk PE. The RCTs included were of good quality but the drawback was that several of them were small sized. Ongoing large scale RCTs will provide further insight and will help shape future guidelines.

## Conclusion

In our meta-analysis of randomized controlled trials of systemic thrombolysis vs anticoagulation in intermediate risk PE, we did not find any difference in in-hospital mortality or in overall risk of major bleeding. With systemic thrombolysis, we found lower risks for vasopressor use and need for secondary/ rescue thrombolysis and an increased risk of intracranial hemorrhage.

### Supplementary Information

**Additional file 1.**

## Data Availability

All data generated or analysed during this study are included in the published article.
